# Blocking of orexin receptors in the paraventricular nucleus of the thalamus has no effect on the expression of conditioned fear in rats

**DOI:** 10.3389/fnbeh.2015.00161

**Published:** 2015-06-16

**Authors:** Xinwen Dong, Yonghui Li, Gilbert J. Kirouac

**Affiliations:** ^1^Department of Oral Biology, College of Dentistry, Faculty of Health Sciences, University of ManitobaWinnipeg, Manitoba, Canada; ^2^Key Laboratory of Mental Health, Institute of Psychology, Chinese Academy of SciencesBeijing, China; ^3^Department of Psychiatry, College of Medicine, Faculty of Health Sciences, University of ManitobaWinnipeg, Manitoba, Canada

**Keywords:** paraventricular nucleus, fear, orexin, learning, midline thalamus

## Abstract

The paraventricular nucleus of the thalamus (PVT) projects to the central nucleus of the amygdala and recent experimental evidence indicates a role for the PVT in conditioned fear. Furthermore, the PVT contains a high density of orexin receptors and fibers and acute injections of orexin antagonist into the PVT produce anxiolytic effects. The present study was done to determine if administration of a dual orexin receptor antagonist (DORA) in the region of the PVT interferes with the expression of conditioned fear in rats exposed to cued and contextual conditioning paradigms. Infusion of 0.5 μl of the DORA N-biphenyl-2-yl-1-[(1-methyl-1H-benzimidazol-2yl) sulfanyl] acetyl-L-prolinamide at a concentration of 0.1, 1.0, and 10 nmol had no effect on the freezing produced by exposing rats to an auditory cue or the context associated with foot shock. In contrast, the 1.0 and 10 nmol doses were anxiolytic in the social interaction test. The results of the present study do not support a role for orexin receptors in the PVT in the expression of learned fear. The finding that the 1.0 and 10 nmol doses of DORA in the PVT region were anxiolytic in the social interaction test is consistent with other studies indicating a role for orexins in the PVT in anxiety-like behaviors.

## Introduction

Orexins (hypocretins), which consist of the bioactive orexin-A and orexin-B peptides, are synthesized exclusively in neurons in the hypothalamus. These peptides act at G-protein coupled receptors called the orexin-1 (OX1R) and the orexin-2 receptors (OX2R). The orexin peptides play a key role in maintaining aroused states and accumulating evidence demonstrates that orexins are involved in the regulation of motivated behavior including food intake and drug seeking (Boutrel et al., 2010; Mahler et al., 2014; Sakurai, 2014). Other evidence shows that orexin neurons are activated in response to stressful conditions (Winsky-Sommerer et al., 2004) and that orexins contribute to anxiety-like behaviors. For instance, systemic injections of the OX1R antagonist SB334867 blocked the behavioral and autonomic responses elicited in a rat model of panic (Johnson et al., 2010). In other studies, acute systemic injections of the dual orexin receptor antagonist (DORA) TCS-1102 reduced contextual fear as well as the defensive and avoidance behaviors in rats previously exposed to footshocks (Chen et al., 2014b). In other studies, chronic oral administration of another DORA called almorexant reduced anxiety after footshock exposure (Viviani et al., 2015) while an acute oral treatment with almorexant attenuated fear in a fear-potentiated startle paradigm (Steiner et al., 2012). The location where the DORA acts to reduce fear is poorly understood but recent reports have identified the locus coeruleus as a brain area where orexins are involved in the acquisition of cue-dependent fear memory (Sears et al., 2013; Soya et al., 2013).

The paraventricular nucleus of the midline thalamus (PVT) is a midline thalamic nucleus that receives among the densest orexin inputs in the brain and represents an area where orexins can act to regulate defensive behaviors. The PVT sends a dense projection to the central nucleus of the amygdala (CeA) and the bed nucleus of the stria terminalis (BST; Li and Kirouac, 2008; Vertes and Hoover, 2008), areas of the brain that play a key role in mediating fear and anxiety (Wilensky et al., 2006; Davis et al., 2010). Furthermore, the PVT projects to the basolateral amygdala, prelimbic cortex and infralimbic cortex (Li and Kirouac, 2008; Vertes and Hoover, 2008), which are known to regulate emotional reactions like fear and anxiety (Burgos-Robles et al., 2009; Sotres-Bayon and Quirk, 2010). More importantly, administrations of orexins in the posterior aspect of the PVT (pPVT) was shown to increase anxiety whereas injections of the OX2R antagonist TCSOX229 was reported to have an acute anxiolytic effect in rats after a previous exposure to footshock stress (Li et al., 2010a,b; Heydendael et al., 2011). Experimental evidence provided from studies using lesion or temporal inactivation in the PVT region also showed that the PVT is involved in the expression of fear to a tone previously paired with footshocks (Padilla-Coreano et al., 2012; Li et al., 2014; Do-Monte et al., 2015; Penzo et al., 2015). Optogenetics or chemogenetics approaches indicated that neurons in the PVT regulated fear expression by a projection to the central amygdala (Do-Monte et al., 2015; Penzo et al., 2015). However, the contribution of orexins in the PVT to conditioned fear has not been investigated.

The present study investigated the effect of blocking orexin receptors in the PVT on the fear associated with cued and contextual conditioning procedures. The purpose of the first experiment was to determine if orexin receptors in the PVT contribute to fear to an auditory tone produced by a conditioning protocol similar to the one used to show an involvement of the PVT in fear (Padilla-Coreano et al., 2012; Li et al., 2014; Do-Monte et al., 2015). The second experiment was done to examine the contribution of orexin receptors in the PVT to the fear associated with the shock chamber using a contextual fear protocol known to cause the activation of the orexin system (Chen et al., 2014a,b). Consequently, the effect of blocking orexin receptors in the PVT on the anxiety that resulted from the contextual conditioning procedure was used to determine the effectiveness of orexin antagonist used in the present experiment.

## Methods

### Animals and Housing

A total of 95 adult male Sprague-Dawley rats (Vital River Laboratory Animal Technology Co. Ltd., Beijing, China) were used for the experiments. The rats (weighing 200–220 g upon arrival) were housed individually in cages with food and water *ad libitum* in a room set on a 12 h/12 h light/dark (lights on at 08:00) cycle with controlled temperature (20–24°C) and humidity (40–70%). The rats were handled for 2 min on alternate days during a 7-day adaption period. All the procedures were conducted according to the National Institutes of Health Guide for the Care and Use of Laboratory Animals. The protocols were approved by the Research Ethics Committee of Institute of Psychology, Chinese Academy of Sciences.

### Surgery

The rats were anesthetized with sodium pentobarbital (60 mg/kg, 2 ml/kg, i.p.; Sigma-Aldrich) and placed in a stereotaxic frame (RWD Life Science Co., Ltd., Shenzhen, Guangdong, China). Stainless steel guide cannulas (23 gauge, RWD Life Science Co., Ltd.) were unilaterally implanted into the posterior aspect of the PVT (3.1 mm posterior to bregma, 1.3 mm lateral to the midline, and 4.0 mm ventral to the skull, angled at 10° with the incisor bar set at 3.3 mm below intraaural line). The guide cannulas were secured with three small screws attached to the skull and dental cement. Capped stylets were inserted into the guide cannulas and all rats were treated with penicillin (80,000 units) to prevent infection. The rats were allowed to recover for 7–10 days and were handled every other day to reduce stress associated with handling.

### Drugs and Microinjections

The DORA used for the experiments was a compound called N-biphenyl-2-yl-1-[(1-methyl-1H-benzimidazol-2yl) sulfanyl] acetyl-L-prolinamide which was first prepared and describe by Merck Research Laboratories (Bergman et al., 2008; TCS-1102, cat# 3818; MW = 470.59, Tocris, Minneapolis, MN, USA) which was dissolved in poly-ethylene glycol 200 (PEG200, Beijing Chemical Works, Beijing, China). TCS-1102 blocks both OX1R (IC_50_ = 4 nM) and OX2R (IC_50_ = 17 nM) and has been shown to be effective in blocking orexin-mediated behaviors for up to 4 h (Bergman et al., 2008; Winrow et al., 2010). The drug and vehicle were microinjected into the PVT (0.5 μl) through an injector cannula (28 gauge, RWD Life Science Co., Ltd.) which protrudes 2.0 mm below the guide cannula. Infusions were delivered with a 5.0 μl Hamilton microsyringe connected to the injector cannula with polyethylene tubing and an infusion pump was used to deliver the drug at the rate of 0.2 μl/min over 2.5 min. The injector cannula was kept in the guide cannula for another minute to prevent backflow before the stylet was placed back into the guide cannula and the rat was returned to its home cage.

### Footshock and Behavioral Procedures

Footshocks were given in an acrylic black chamber (30 × 30 × 27 cm) with a grid floor (Beijing MacroAmbition S&T Development Co., Ltd., Beijing, China) which was illuminated at 200–300 lux. The rats were acclimated to the shock chamber for 180 s before receiving a series of footshocks.

Experiment 1. A cued fear conditioning protocol was used in which rats were exposed to a total of eight 30 s tones (4 kHz, 75 dB) that co-terminated with a 0.5 s shock of 0.65 mA (inter trial interval = 120 ± 30 s). The rats were assigned to four homogeneous groups based on their freezing time during fear learning. Fear expression to the tone was tested 24 h later in rats that had received a microinjection of the DORA into the PVT region approximately 30 min before being placed in the same chamber that was used for conditioning. The rats received the DORA at 0.1, 1.0, and 10 nmol or the vehicle. Each rat was exposed to the tone 20 times (inter trial interval of 60 ± 30 s). Fear extinction recall was examined 24 h later by exposing the rats to 10 tones in the shock chamber. The purpose of the extinction recall experiment was to examine if the DORA interfered with the consolidation processes of the extinction memory. The freezing response, which was defined as no movement except for those related to respiration, was recorded by a video tracking system (XeyeFCs, Beijing MacroAmbition S&T Development Co., Ltd., Beijing, China) and was used as a measure of fear.

Experiment 2. A contextual fear conditioning protocol was used in which rats were exposed to five 5 s footshocks of 1.5 mA (intershock interval of 120 ± 30 s). Animals were returned to their home cages 60 s after the last footshock. The choice to use more intense footshocks for the contextual conditioning experiment was based on previous evidence that exposure of 1.5 mA footshocks produces an orexin-mediated fear and anxiety (Chen et al., 2014b). For example, exposure of rats to footshocks of this intensity has been shown to produce long-lasting increases in prepro-orexin mRNA as well as contextual fear and anxiety that are attenuated by systemic injections of a DORA (Chen et al., 2014b). Rats were assigned to drug treatment groups as described for Experiment 1. Fear expression was examined 24 h later by placing the rat in the shock chamber for 10 min following microinjections of the drug in the PVT region. Fourteen days later, the animals were reassigned to four groups randomly and microinjections of one of three different doses of the DORA or the vehicle were made in the PVT region before the behavior of the rats was examined consecutively in the open field and social interaction tests. Note that TCS 1102 was shown to block orexin receptors for at least 4 h after administration (Winrow et al., 2010). Thirty minutes later, the rats were placed in a circular open field (100 cm in diameter, 50 cm high, with 15–20 lux illumination) for 5 min. The activity of the rats was recorded by the video tracking system for subsequent analysis of locomotor activity, latency to the center area, and time spent in the center area. After the open field test, the rats were transferred to another room for the social interaction test. The social interaction arena was a black rectangle open field (100 cm long × 60 cm wide, 5–10 lux illumination). A stimulus rat (male rat weighing 450–500 g) was put into a small mesh box in a corner of the arena, and the test rat was placed in an enclosed compartment located in the opposite corner. After a 2 min habituation period, the sliding door of the enclosed box was opened, allowing the test rat to move freely in the arena for 5 min. The time spent in the social zone (20 × 30 cm zone immediately adjacent to the target rat compartment) and the latency to come out of the box was recorded by the video tracking system.

### Cannula Verification

**Figure 1:**
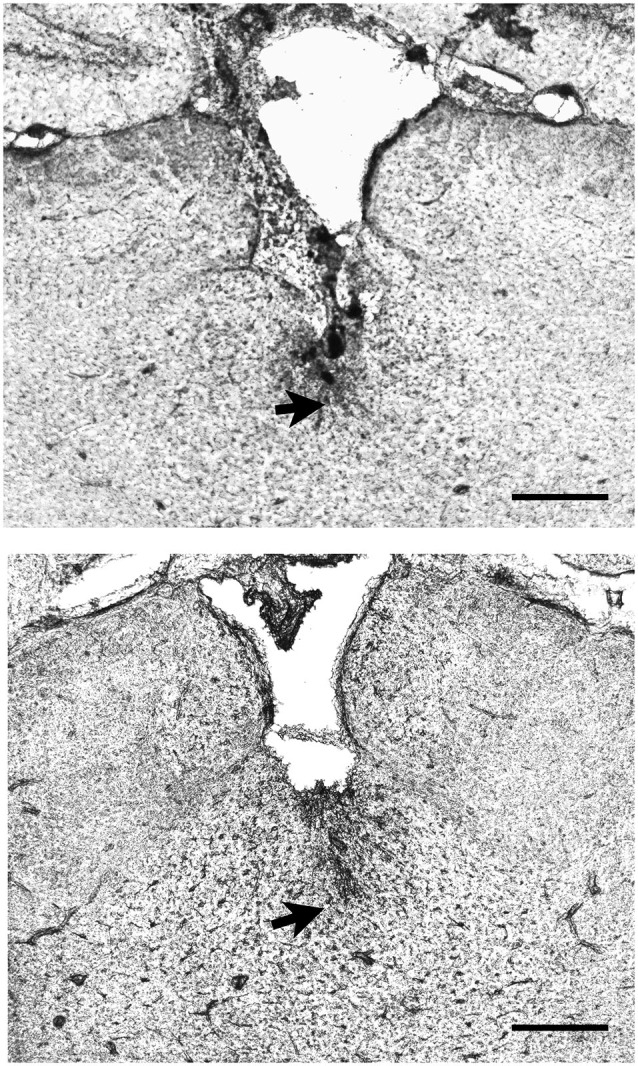
**Examples of the location of injector tips in the thalamus**. The pictures show two examples of placements of the injector tip (arrows) in the region of the paraventricular nucleus of the thalamus (PVT) as verified from histological sections. Scale bar = 0.5 mm.

**Figure 2:**
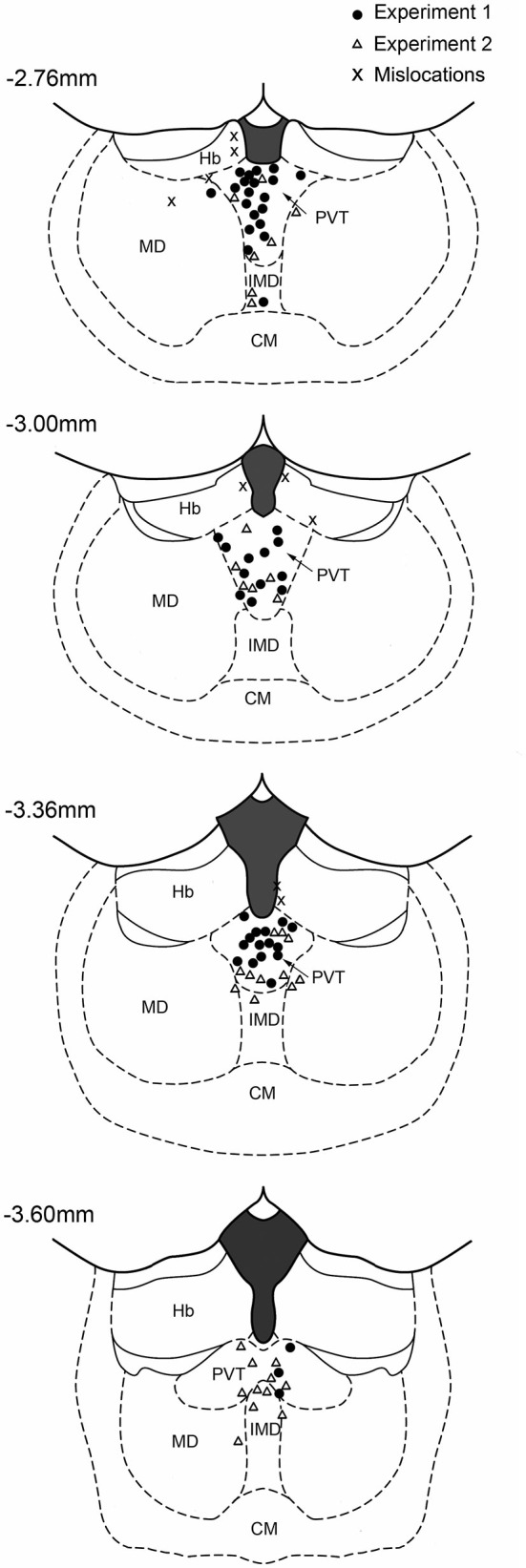
**Location of injector cannulas in the midline thalamus**. The diagram shows the location of the injector tips in the region of the PVT as verified from histological sections. The numbers correspond to distance from bregma. CM, centromedial nucleus; IMD, intermediodorsal nucleus; Hb, habenular complex; MD, mediodorsal nucleus.

Rats were deeply anesthetized with chloral hydrate (50 mg/kg) prior to decapitation. The brains were removed, post-fixed overnight in 4% paraformaldehyde and subsequently cryoprotected in 30% sucrose solution in 0.1 M phosphate buffer (PB) at 4°C. Serial coronal sections (60 μm) were cut using a freezing microtome and subsequently stained for Nissl substance using Cresyl Violet. Two examples of cannula placement are shown in Figure 1 and the locations for all cases in the two experiments are shown in Figure 2. Data of rats with misplaced cannula were excluded (three in Experiment 1, six in Experiment 2), only rats with injection sites near the PVT were used for statistical analysis (Experiment 1: 0.1 nmol *n* = 13, 1.0 nmol *n* = 12, 10 nmol *n* = 11, vehicle *n* = 14; Experiment 2: 0.1 nmol *n* = 7, 1.0 nmol *n* = 10, 10 nmol *n* = 10, vehicle *n* = 9; Experiment 2 reassignment for anxiety tests 0.1 nmol *n* = 10, 1.0 nmol *n* = 7, 10 nmol *n* = 10, vehicle *n* = 9).

### Statistical Analysis

The freezing responses during the presentation of the tone for the extinction and extinction recall sessions (Experiment 1; freezing for each trial consisting of two consecutive tone presentations) as well as the freezing to the shock chamber (Experiment 2; each data point consisting of a 2.5 min time period) were analyzed using an ANOVA with repeated-measures. For Experiment 2, the total amount of freezing in the shock chamber, locomotion in the open field and time spent in social zone were analyzed by one-way ANOVA with Bonferroni *post hoc* tests used for multiple comparisons. The latency in the open field test and in the social interaction test were analyzed by Kruskal-Wallis one-way analysis of variance by ranks followed by Mann-Whitney test to determine which group was statistically different. Statistical analysis was done with SPSS 20.0 software and data are presented as the mean ± standard error of the mean.

## Results

### Experiment 1

**Figure 3:**
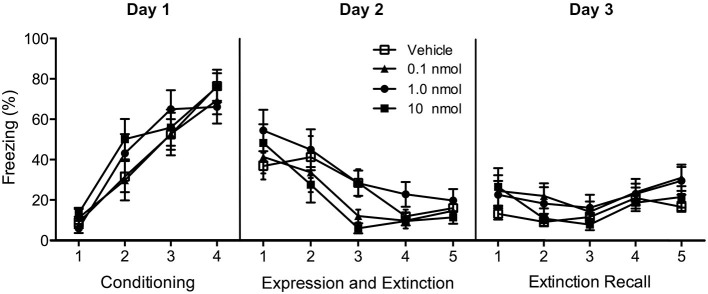
**Effect of injections of a DUAL OREXIN RECEPTOR ANTAGONIST (DORA) in the region of the PVT on freezing in cued fear conditioning**. Rats were fear conditioned on Day 1 and freezing to the tone was examined on Day 2 following injections of the vehicle or one of the 0.1, 1.0, and 10 nmol concentrations of the DORA. The drug had no effect on fear expression or fear extinction. In addition, the drug treatment on Day 2 had no effect on the extinction recall on Day 3. Each data point represents the mean of two trials and results are presented as mean ± SEM.

In Experiment 1, exposure of rats to tones paired with footshocks resulted in increasing amounts of freezing with conditioning trials (*F*_(3,138)_ = 68.3, *p* < 0.001; Figure 3). Repeated presentation of the conditioning tone produced a significant extinction effect (main effect of extinction in repeated-measures ANOVA *F*_(9,414)_ = 13.3, *p* < 0.001; Figure 3) and the DORA did not affect fear expression during the first tone presentation (*F*_(3,46)_ = 0.51, *p* = 0.66; Figure 3) nor the fear extinction in the extinction trials that followed (*F*_(3,46)_ = 1.68, *p* = 0.18). In addition, there was no interaction between the trials and DORA doses *F*_(27,414)_ = 1.13, *p* = 0.30; Figure 3). The pre-tone baseline level of freezing in the chamber was unaffected by the DORA (vehicle 21.7%, 0.1 nmol 26.9%, 1.0 nmol 21.2%, and 10 nmol 20.7%; *F*_(3,46)_ = 0.17, *p* = 0.92). There were no group differences in the recall of the extinction memory 24 h after the extinction phase (*F*_(3,46)_ = 1.512, *p* = 0.22; Figure 3), nor was there an interaction between doses of DORA and the trials (*F*_(12,184)_ = 0.70, *p* = 0.75; Figure 3).

### Experiment 2

**Figure 4:**
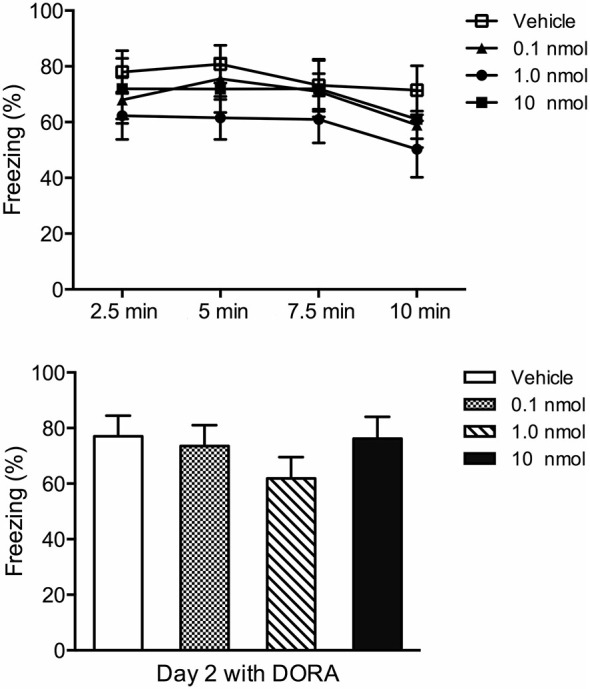
**Effect of injections of a DORA in the PVT region in contextual fear conditioning**. Rats were exposed to footshock and the amount of freezing to the shock chamber was examined on Day 2 following injections of the vehicle or one of the 0.1, 1.0, and 10 nmol concentration of the DORA. The drug had no effect on fear expression. The results are presented as mean ± SEM.

**Figure 5:**
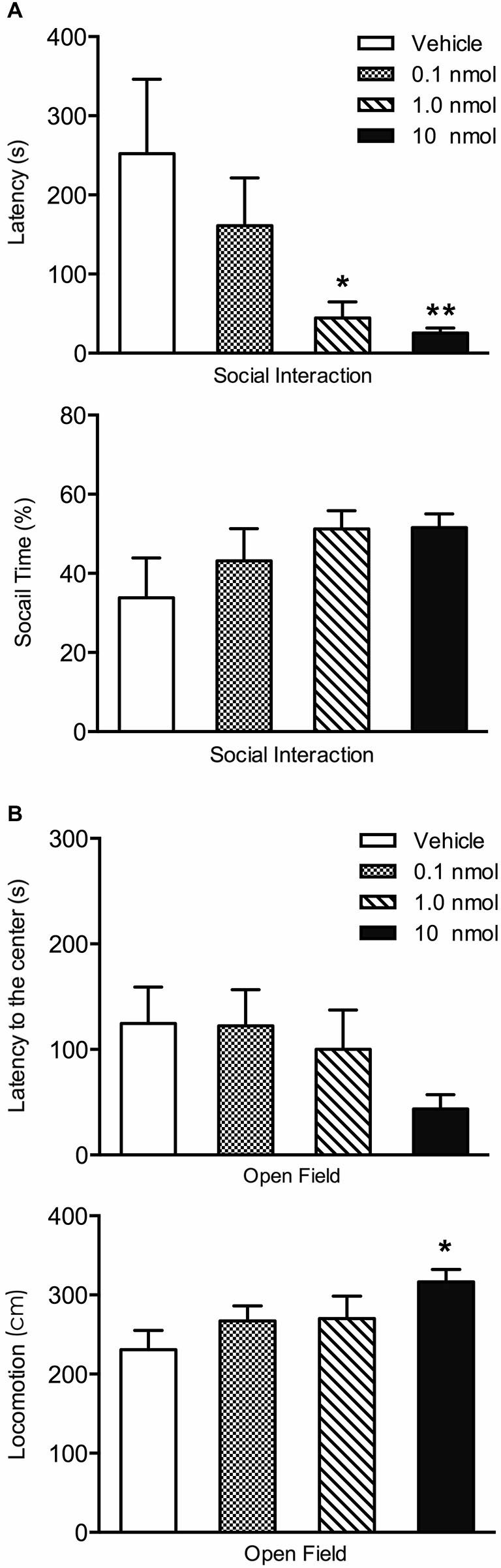
**Effect of injections of a DORA in the region of the PVT on avoidance. (A)** The 1.0 and 10 nmol concentrations of the DORA decreased the latency for rats to enter the social zone in the social interaction test while the same doses did not significantly affect the time spent in the social zone. **(B)** Injections of the DORA did not have a significant effect on the latency to enter the center of the open field while the 10 nmol concentration increased the amount of locomotion.Note that injections of the DORA were made in the PVT prior to exposing the rats consecutively to the open field and social interaction tests. *p < 0.05; **p < 0.01. Results are presented as mean ± SEM.

A contextual fear conditioning paradigm using footshocks with an intensity of 1.5 mA produces fear to the shock context (contextual fear condition) and anxiety in tests involving approach-avoidance behaviors (Chen et al., 2012). Consequently, we used this paradigm to investigate the role of orexin receptors in the PVT in fear and anxiety. In terms of the contextual fear expression 24 h after fear conditioning, the DORA had no effect on fear to the shock chamber (freezing in four 2.5 min time periods *F*_(3,32)_ = 0.72, *p* = 0.55, interaction between time and DORA doses *F*_(9,96)_ = 0.15, *p* = 0.99; total freezing for the 10 min in the shock chamber *F*_(3,32)_ = 1.01, *p* = 0.40; Figure 4). However, in the social interaction test, the Kruskal-Wallis one-way ANOVA indicated a main effect for the DORA ($\chi_{\left(3\right)}^{2}$ = 11.78, *p* = 0.008; Figure 5A) with the 1.0 nmol (*p* = 0.038) and 10 nmol (*p* = 0.003) doses decreasing the latency to enter the social interaction zone. The time spent in the social zone was not significantly increased in groups with DORA (*F*_(3,32)_ = 1.47, *p* = 0.24; Figure 5A). In the open field test, the DORA had no effect on the latency to go to the center ($\chi_{\left(3\right)}^{2}$ = 5.55, *p* = 0.14; Figure 5B) but the one-way ANOVA revealed a main effect of drug dose on locomotion (*F*_(3,32)_ = 3.27, *p* = 0.034; Figure 5B) with the highest dose significantly increasing locomotion (*p* = 0.024). In summary, the DORA had an anxiolytic effect but had no effect on contextual fear expression.

## Discussion

The present study investigated the role of orexin receptors in the PVT in cued and contextual fear conditioning. The results show that microinjections of a DORA in the PVT did not attenuate the fear expressed following exposure of rats to cued and contextual fear conditioning paradigms. A lack of an effect on contextual fear was surprising considering that the protocol used was the same as the one used to show that systemic injections of the same DORA reduced contextual fear (Chen et al., 2014b). However, the DORA had an anxiolytic effect when the contextual fear conditioned rats were tested in a social interaction test. These results indicate that orexin receptors in the PVT are not involved in learned fear expression but play a role in anxiety.

A number of studies have shown that the PVT is involved in the expression of a learned fear or the retrieval of a fear memory in the cued conditioning model (Padilla-Coreano et al., 2012; Li et al., 2014; Do-Monte et al., 2015; Penzo et al., 2015) while other studies indicate a role for orexin receptors in the PVT in anxiety (Li et al., 2010a,b; Heydendael et al., 2011). As such, we tested the possibility that orexin receptors in the PVT were involved in learned fear. However, the results of the experiments indicate that orexin receptors in the PVT are not necessary for the expression of cued fear memory. This conclusion is consistent with the previous experimental evidence that demonstrated that the orexin system is not involved in cued fear expression or memory recall (Sears et al., 2013; Soya et al., 2013). For example, cued fear expression was reported to be unaffected by a prior intracerebroventricular (ICV) infusion of the OX1R antagonist SB334867 in rats trained using a weak training protocol (3 × 0.5 mA 2 s footshocks; Sears et al., 2013). In addition, OX2R knock-out mice display a similar level of fear learning and expression as wild type animals when they are exposed to a cued fear conditioning paradigm with mild footshocks (Soya et al., 2013). Taken together, it does not appear that the orexin system contributes to the freezing associated with discriminative fear conditioning. It is possible that the orexin system is involved in other types of response associated with discriminative fear paradigms since other studies have reported that an acute oral administration of another DORA attenuated a conditioned fear potentiated startle response (Steiner et al., 2012). In that study the conditioning training session contained more trials (15 pairs each session) and trained the rats two or four times before the fear potentiated startle test. The relatively prolonged training session might result in a more robust fear memory possibly through mechanisms involving the orexin system. In addition, freezing and the startle response are incompatible behavioral responses associated with fear (Davis and Astrachan, 1978). Since the startle response is often considered a measure of arousal (Lang and Davis, 2006), it is possible that arousal peptides like orexins may be preferentially involved in fear potential startle.

Exposure of rats to a single and brief episode of moderately intense footshocks (1.5 mA) results in an increase in the levels of prepro-orexin mRNA in the hypothalamus and systemic treatment of shocked rats with a DORA reduce contextual fear and the anxiety displayed by shocked rats tested in the elevated T maze (Chen et al., 2014b). In this study, we tested the hypothesis that blocking of orexin receptors in the PVT would attenuate the expression of contextual fear. This hypothesis was based on findings that blocking of OX2R in the PVT decreased the anxiety of rats tested in the elevated plus maze and made anxious by exposure to one 2.0 mA footshock for 10 s 2 days earlier (Li et al., 2010b). Contrary to our hypothesis, injections of the DORA in the PVT region had no effect on contextual fear. The anxiety tests in experiment 2 were conducted to verify that the microinjections of the DORA blocked orexin receptors in the PVT. Indeed, the moderate and high doses of the DORA had anxiolytic effects in the social interaction test. These results indicate that orexin receptors in the PVT are not involved in contextual fear but that these receptors play a role in regulating anxiety. Our observation that injection of the highest dose of the DORA in the PVT had a trend towards being anxiolytic when rats were tested in the open field suggests a preferential recruitment of orexin receptors in the PVT in social anxiety. The anxiolytic effects of blocking orexin receptors in the PVT have been previously reported (Li et al., 2010b), which is consistent with similar anxiolytic effects reported when orexin antagonists are administered via systemic routes (Chen et al., 2014b; Viviani et al., 2015). In these experiments, exposure of rats to relatively high footshock intensity (1.5–2.0 mA) led to an orexin-mediated anxiety. The fact that contextual fear learning was impaired in knockout mice and that systemic injection of a DORA attenuated some measures of contextual fear (Furlong et al., 2009; Chen et al., 2014b) indicates that orexins are involved in contextual fear but not through actions on orexin receptors in the PVT.

These findings should also be viewed from the perspective that other research showing that blocking of orexin receptors in the PVT has anxiolytic effect (Li et al., 2010a,b; Heydendael et al., 2011). A dose dependent decrease in the latency in the social interaction test indicates that microinjections of the DORA in the PVT produced a previously characterized pharmacological effect for orexin receptors in this area of the thalamus. As such, the negative results associated with the fear conditioning experiments in the present study are unlikely due to inadequate antagonism of orexin receptors in the PVT. The fact that injections of a DORA in the PVT attenuated anxiety while having no effect on learned fear also suggests that activation of orexin receptors in the PVT leads to a recruitment of brain pathways or mechanisms related to anxiety.

## Conflict of Interest Statement

The authors declare that the research was conducted in the absence of any commercial or financial relationships that could be construed as a potential conflict of interest.

## References

[B1] Bergman J. M. Roecker A. J. Mercer S. P. Bednar R. A. Reiss D. R. Ransom R. W.. (2008). Proline bis-amides as potent dual orexin receptor antagonists. Bioorg. Med. Chem. Lett. 18, 1425–1430. 10.1016/j.bmcl.2008.01.001 18207395

[B2] Boutrel B. Cannella N. de Lecea L. (2010). The role of hypocretin in driving arousal and goal-oriented behaviors. Brain Res. 1314, 103–111. 10.1016/j.brainres.2009.11.054 19948148 PMC4307927

[B3] Burgos-Robles A. Vidal-Gonzalez I. Quirk G. J. (2009). Sustained conditioned responses in prelimbic prefrontal neurons are correlated with fear expression and extinction failure. J. Neurosci. 29, 8474–8482. 10.1523/jneurosci.0378-09.2009 19571138 PMC2733220

[B30] Chen X. Li Y. Li S. Kirouac G. J. (2012). Early fear as a predictor of avoidance in a rat model of post-traumatic stress disorder. Behav. Brain Res. 226, 112–117. 10.1016/j.bbr.2011.09.004 21924297

[B4] Chen X. Li S. Kirouac G. J. (2014a). Blocking of corticotrophin releasing factor receptor-1 during footshock attenuates context fear but not the upregulation of prepro-orexin mRNA in rats. Pharmacol. Biochem. Behav. 120, 1–6. 10.1016/j.pbb.2014.01.013 24491435

[B5] Chen X. Wang H. Lin Z. Li S. Li Y. Bergen H. T.. (2014b). Orexins (hypocretins) contribute to fear and avoidance in rats exposed to a single episode of footshocks. Brain Struct. Funct. 219, 2103–2118. 10.1007/s00429-013-0626-3 23955372

[B6] Davis M. Astrachan D. I. (1978). Conditioned fear and startle magnitude: effects of different footshock or backshock intensities used in training. J. Exp. Psychol. Anim. Behav. Process. 4, 95–103. 10.1037/0097-7403.4.2.95 670892

[B7] Davis M. Walker D. L. Miles L. Grillon C. (2010). Phasic vs. sustained fear in rats and humans: role of the extended amygdala in fear vs. anxiety. Neuropsychopharmacology 35, 105–135. 10.1038/npp.2009.109 19693004 PMC2795099

[B8] Do-Monte F. H. Quiñones-Laracuente K. Quirk G. J. (2015). A temporal shift in the circuits mediating retrieval of fear memory. Nature 519, 460–463. 10.1038/nature14030 25600268 PMC4376623

[B9] Furlong T. M. Vianna D. M. Liu L. Carrive P. (2009). Hypocretin/orexin contributes to the expression of some but not all forms of stress and arousal. Eur. J. Neurosci. 30, 1603–1614. 10.1111/j.1460-9568.2009.06952.x 19811530

[B10] Heydendael W. Sharma K. Iyer V. Luz S. Piel D. Beck S.. (2011). Orexins/hypocretins act in the posterior paraventricular thalamic nucleus during repeated stress to regulate facilitation to novel stress. Endocrinology 152, 4738–4752. 10.1210/en.2011-1652 21971160 PMC3230061

[B11] Johnson P. L. Truitt W. Fitz S. D. Minick P. E. Dietrich A. Sanghani S.. (2010). A key role for orexin in panic anxiety. Nat. Med. 16, 111–115. 10.1038/nm.2075 20037593 PMC2832844

[B12] Lang P. J. Davis M. (2006). Emotion, motivation and the brain: reflex foundations in animal and human research. Prog. Brain Res. 156, 3–29. 10.1016/s0079-6123(06)56001-7 17015072

[B13] Li Y. Dong X. Li S. Kirouac G. J. (2014). Lesions of the posterior paraventricular nucleus of the thalamus attenuate fear expression. Front. Behav. Neurosci. 8:94. 10.3389/fnbeh.2014.00094 24688461 PMC3960725

[B14] Li S. Kirouac G. J. (2008). Projections from the paraventricular nucleus of the thalamus to the forebrain, with special emphasis on the extended amygdala. J. Comp. Neurol. 506, 263–287. 10.1002/cne.21502 18022956

[B15] Li Y. Li S. Wei C. Wang H. Sui N. Kirouac G. J. (2010a). Changes in emotional behavior produced by orexin microinjections in the paraventricular nucleus of the thalamus. Pharmacol. Biochem. Behav. 95, 121–128. 10.1016/j.pbb.2009.12.016 20045021

[B16] Li Y. Li S. Wei C. Wang H. Sui N. Kirouac G. J. (2010b). Orexins in the paraventricular nucleus of the thalamus mediate anxiety-like responses in rats. Psychopharmacology (Berl) 212, 251–265. 10.1007/s00213-010-1948-y 20645079

[B17] Mahler S. V. Moorman D. E. Smith R. J. James M. H. Aston-Jones G. (2014). Motivational activation: a unifying hypothesis of orexin/hypocretin function. Nat. Neurosci. 17, 1298–1303. 10.1038/nn.3810 25254979 PMC4335648

[B18] Padilla-Coreano N. Do-Monte F. H. Quirk G. J. (2012). A time-dependent role of midline thalamic nuclei in the retrieval of fear memory. Neuropharmacology 62, 457–463. 10.1016/j.neuropharm.2011.08.037 21903111 PMC3195904

[B19] Penzo M. A. Robert V. Tucciarone J. De Bundel D. Wang M. Van Aelst L.. (2015). The paraventricular thalamus controls a central amygdala fear circuit. Nature 519, 455–459. 10.1038/nature13978 25600269 PMC4376633

[B20] Sakurai T. (2014). The role of orexin in motivated behaviours. Nat. Rev. Neurosci. 15, 719–731. 10.1038/nrn3837 25301357

[B21] Sears R. M. Fink A. E. Wigestrand M. B. Farb C. R. de Lecea L. Ledoux J. E. (2013). Orexin/hypocretin system modulates amygdala-dependent threat learning through the locus coeruleus. Proc. Natl. Acad. Sci. U S A 110, 20260–20265. 10.1073/pnas.1320325110 24277819 PMC3864341

[B22] Sotres-Bayon F. Quirk G. J. (2010). Prefrontal control of fear: more than just extinction. Curr. Opin. Neurobiol. 20, 231–235. 10.1016/j.conb.2010.02.005 20303254 PMC2878722

[B23] Soya S. Shoji H. Hasegawa E. Hondo M. Miyakawa T. Yanagisawa M.. (2013). Orexin receptor-1 in the locus coeruleus plays an important role in cue-dependent fear memory consolidation. J. Neurosci. 33, 14549–14557. 10.1523/jneurosci.1130-13.2013 24005305 PMC6618384

[B24] Steiner M. A. Lecourt H. Jenck F. (2012). The brain orexin system and almorexant in fear-conditioned startle reactions in the rat. Psychopharmacology (Berl) 223, 465–475. 10.1007/s00213-012-2736-7 22592903

[B25] Vertes R. P. Hoover W. B. (2008). Projections of the paraventricular and paratenial nuclei of the dorsal midline thalamus in the rat. J. Comp. Neurol. 508, 212–237. 10.1002/cne.21679 18311787

[B26] Viviani D. Haegler P. Jenck F. Steiner M. A. (2015). Orexin neuropeptides contribute to the development and persistence of generalized avoidance behavior in the rat. Psychopharmacology (Berl) 232, 1383–1393. 10.1007/s00213-014-3769-x 25319964

[B27] Wilensky A. E. Schafe G. E. Kristensen M. P. LeDoux J. E. (2006). Rethinking the fear circuit: the central nucleus of the amygdala is required for the acquisition, consolidation and expression of Pavlovian fear conditioning. J. Neurosci. 26, 12387–12396. 10.1523/jneurosci.4316-06.2006 17135400 PMC6674909

[B28] Winrow C. J. Tanis K. Q. Reiss D. R. Rigby A. M. Uslaner J. M. Uebele V. N.. (2010). Orexin receptor antagonism prevents transcriptional and behavioral plasticity resulting from stimulant exposure. Neuropharmacology 58, 185–194. 10.1016/j.neuropharm.2009.07.008 19596018

[B29] Winsky-Sommerer R. Yamanaka A. Diano S. Borok E. Roberts A. J. Sakurai T.. (2004). Interaction between the corticotropin-releasing factor system and hypocretins (orexins): a novel circuit mediating stress response. J. Neurosci. 24, 11439–11448. 10.1523/jneurosci.3459-04.2004 15601950 PMC6730356

